# Evaluation of Predictive Factors for Successful Intravitreal Dexamethasone in Pseudophakic Cystoid Macular Edema

**DOI:** 10.1155/2017/4625730

**Published:** 2017-12-19

**Authors:** Vinodh Kakkassery, Tim Schultz, Marc Ilan Wunderlich, Marc Schargus, H. Burkhard Dick, Jörg Rehrmann

**Affiliations:** ^1^Department of Ophthalmology, Ruhr-University, Bochum, Germany; ^2^Department of Ophthalmology, University of Rostock, Rostock, Germany; ^3^Department of Ophthalmology, Heinrich Heine University, Duesseldorf, Germany

## Abstract

**Purpose:**

To determine the efficacy, safety, and predictive outcome factors for intravitreal dexamethasone implant (DEX) in pseudophakic cystoid macular edema (PCME).

**Methods:**

Retrospective, interventional, controlled study. Patients included had to have clinically significant PCME and have been treated with the DEX between 2012 and 2015. Charts and one-year data were selected consecutively, and efficacy and safety were abstracted. Visual acuity (VA) and central foveal thickness (CFT) were analysed.

**Results:**

Nineteen patient data sets were analysed. After treatment with DEX, mean VA increased significantly by 0.2 logMAR (*p* = 0.034), while the mean CFT was reduced significantly by 162.79 *μ*m (*p* < 0.001). Five patients receiving a combination of DEX/bevacizumab have not experienced a higher mean VA gain or CFT reduction compared to fourteen patients receiving DEX alone. Decision rules, when to combine DEX with bevacizumab, have not been defined before the study. Only posttreatment VA gains in the nonhypertensive subgroup (*n* = 11) were significantly better (*p* = 0.026). Analysis of data from diabetes patients (*n* = 4) versus nondiabetics yielded no significant differences in efficacy. There have been no adverse events within follow-up time.

**Conclusion:**

The use of DEX in PCME showed significant improvements in VA and CFT. The VA seems to show greater improvements in patients without hypertension.

## 1. Introduction

Pseudophakic cystoid macular edema (PCME) is characterized by fovea swelling due to fluid accumulation occurring weeks to months after cataract surgery [1–3]. The incidence of symptomatic PCME ranges from 0.1 to 2.35% and rises with risk factors, such as patient's age, arterial hypertension, and diabetes mellitus [3–6]. Further risk factors have been identified with the method of intraocular lens replacement as well as with complications during surgery [3–5, 7, 8]. The precise pathophysiology of PCME remains uncertain. Different inflammatory mediators have been postulated to be involved in the pathophysiology of PCME [9–13]. The multitude of mechanisms suggested is reflected by many different clinical management approaches, such as topical or systemic nonsteroidal or steroidal anti-inflammatory agents (NSAIDs) [3, 14]. Recurrence of PCME after successful therapy is very low, but still some cases are refractive to standard therapy [15]. Intravitreal dexamethasone (DEX) implant has been introduced as a new therapy strategy in PCME in recent years [16–22]. Mean visual acuity increased, and mean central macular thickness decreased significantly in studies [16–22]. However, further evidence is needed due to the low numbers of PCME cases analysed after a DEX implant.

The objective of this retrospective study is to determine the further efficacy of a DEX implant in PCME and, thus, increase the evidence for successful DEX implant therapy with 19 additional cases. We have seen variability in the PCME response to DEX implants in our analysis. Therefore, the objective of this study is to evaluate diabetes mellitus and arterial hypertension as possible predictive factors for successful DEX implant therapy in PCME.

## 2. Materials and Methods

### 2.1. Study Design and Patients

This study was performed as a retrospective, open-labelled, interventional, uncontrolled study. Data from patients with diagnosed PCME and treated with a DEX implant between 2012 and 2015 were analysed retrospectively. Intravitreal DEX implant treatment had been performed as an off-label approach. Medical informed consent including the off-label-use of DEX implant was performed before treatment. Reimbursement for DEX implant was covered by the hospital. The retrospective chart review adhered to the tenets of the Declaration of Helsinki, and ethics committee approval (Register number 15-5556, Ruhr-University Ethics Committee, Bochum, Germany) was obtained. Medical charts were selected consecutively and screened for study suitability. Data were collected and fully de-identified prior to analysis. Optical coherence tomography (Spectralis OCT, Heidelberg Engineering, Heidelberg, Germany) was performed to measure central foveal thickness (CFT).

Inclusion criteria were age ≥ 18 years, clinically significant and diagnosed PCME (cystoid macular edema with a minimum of 300 *μ*m, refractive to topical or oral medication), administration of a DEX implant, and occurrence of symptoms less than six months after cataract surgery. Exclusion criteria were defined by not accomplishing the inclusion criteria, fibrotic changes, atrophic changes, or visual acuity limiting disease of the macular, steroid-induced glaucoma. Patients with clinical signs for diabetic maculo- or retinopathy and arterial hypertension retinopathy before or/and after treatment have been also excluded. None of the patients suffered from uveitis or had previous intraocular surgery after the cataract surgery.

Arterial hypertension patients were defined as those with medically confirmed diagnosis and arterial hypertension medication. Diabetes mellitus patients were defined as those with medically confirmed diagnosis and oral medication for diabetes mellitus or insulin medication.

### 2.2. DEX Implant in PCME Patients

The DEX implant (dexamethasone 700 *μ*g) single injections were conducted in accordance with the German Ophthalmic Society guidelines for intravitreal injections (http://www.dog.org). The DEX implant treatment administered after PCME had proven to be refractive to treatment with topical NSAID or oral carbonic anhydrase inhibitor. The DEX implant was administered in combination with an intravitreal bevacizumab injection (1.25 mg/0.05 ml) in five patients.

### 2.3. DEX Implant Treatment Success Control and Safety Data Recording

Basic demographic data and relevant clinical outcome parameters were abstracted for a period of twelve months following the first visit to the study centre. This included demographic and anamnestic data, follow-up times, safety findings, and data on efficacy (including visual acuity, central foveal thickness, and time to first treatment with the DEX implant after occurrence of symptoms).

Therapy success was measured as a logarithm of the minimum angle of resolution (logMAR) VA gain and a reduction in CFT. Statistical analysis was carried out for logMAR visual acuity and CFT comparing pre- and posttreatment results. Posttreatment results have been taken from patients' last visit in the clinic. Approximate Snellen equivalents (based on the conversion table published by the German Society of Ophthalmology) are given in parentheses for all mean absolute logMAR values throughout the manuscript.

### 2.4. Analysis

Data are presented as mean values (±SD; max/min values) unless otherwise stated. The groups were compared by ANOVA followed by Tukey post hoc test using Statistica Software (V10.0, Statsoft, Tulsa, OK, USA). *P* values below 0.05 were considered statistically significant.

## 3. Results

### 3.1. Baseline Characteristics

The retrospective study included a total of 19 eyes from 19 patients (seven male, twelve female). The mean age of the patients at cataract surgery was 69 years (±7.9 years). Ten right eyes and nine left eyes were treated. The PCME developed an average of 42 days (±25.8 days) after cataract surgery. All patients had initially received other topical and/or systemic therapies, such as diclofenac eye drops (*n* = 18), prednisolone eye drops (*n* = 1), oral acetazolamide (*n* = 16), and oral prednisolone (*n* = 1). Three patients refused to take any oral medication, due to previous experience with these oral medications. The mean time to DEX implant administration was 195 days (±149 days) after the first symptoms. While 14 patients received DEX implants as monotherapy, five patients received additional injections with intravitreal bevacizumab. We identified the following systemic comorbidities in our cohort: four patients suffered from diabetes while eight patients had arterial hypertension. A summary of demographic and anamnestic data, follow-up times, and safety findings is presented in Tables 1 and 2. Neither ocular nor systemic side effects were observed during the course of treatment.

### 3.2. Total Group Differences Pre- and Postintravitreal DEX Treatment for VA and CFT in PCME

**Figure 1:**
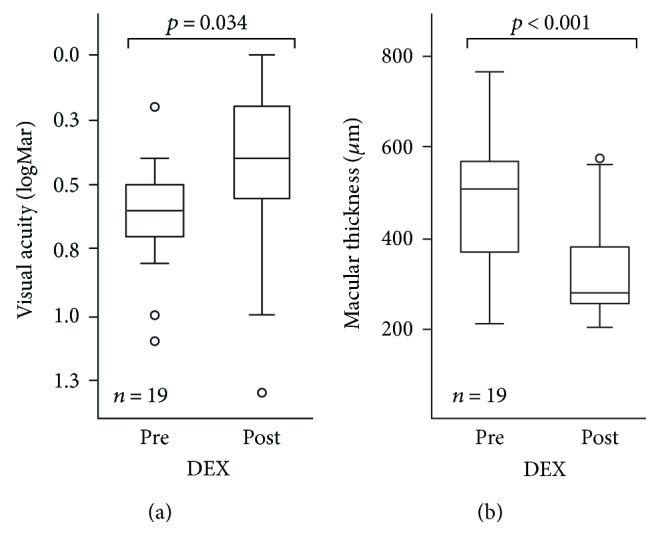
Visual acuity and macular thickness prior to and post DEX implantation. (a) The box plot shows median visual acuity in logMAR (vertical line in the column), 95% confidence interval (column), and the standard deviation (extension lines) before and after treatment with DEX implantation. Statistical analysis with ANOVA followed by a Tukey post hoc test demonstrated a statistically significant increase of visual acuity after treatment with a DEX implant (*p* = 0.034). (b) The box plot shows mean central foveal thickness in *μ*m (vertical line in the column), 95% confidence interval (column), and the standard deviation (extension lines) before and after treatment with DEX implantation. Statistical analysis with ANOVA followed by Tukey post hoc test demonstrated a statistically significant decrease of central foveal thickness after treatment with a DEX implant (*p* < 0.001). ° symbolizes single values with a 1.5- to 3-fold interquartile range distance to the median.

Mean baseline VA of 0.63 ± 0.22 logMAR (Snellen equivalents: 20/80, highest VA: 0.2 logMAR, lowest VA: 1.1 logMAR) increased to 0.43 ± 0.3 logMAR (Snellen equivalents: 20/50, max/min: 0 logMAR/1.3 logMAR) after intravitreal DEX treatment (Figure 1(a)). Mean VA (logMAR) for the total group increased significantly by 0.2 (±0.32, *p* = 0.034).

Mean baseline central foveal thickness (CFT) of 496.42 *μ*m (±150.73, max/min: 767 *μ*m/310 *μ*m) was reduced to 333.60 *μ*m (±103.74, max/min: 574 *μ*m/216 *μ*m) after intravitreal DEX treatment. This reduction of 162.79 *μ*m (±158.43, *p* < 0.001) was also statistically significant (Figure 1(b)).

**Figure 2:**
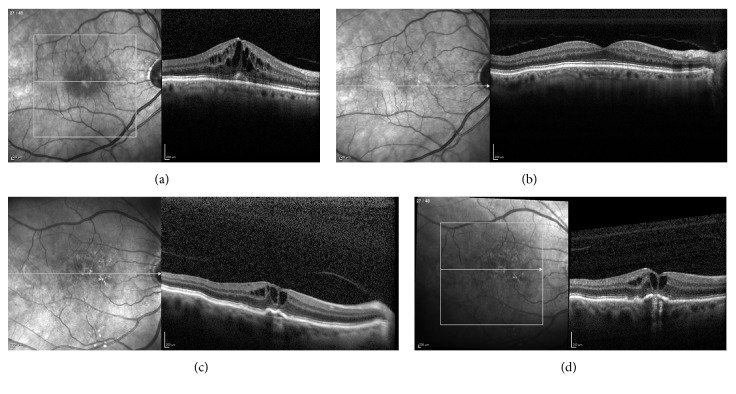
Optical coherence tomography (OCT) examples after DEX implant treatment success and treatment failure. (a) OCT image of a PCME patient (a massive macula edema with cyst, central retinal thickness 522 *μ*m) without diabetes and arterial hypertension before DEX implant therapy and (b) after DEX implant therapy (no longer either a macula edema or cyst, central retinal thickness 285 *μ*m). (c) OCT image of a PCME patient (a macula edema with cyst, central retinal thickness 384 *μ*m) without diabetes, but with arterial hypertension before DEX implant therapy and (d) after DEX implant therapy (a macula edema with cyst similar to pretreatment, central retinal thickness 370 *μ*m).

Taken together, both VA and CFT increased significantly after intravitreal DEX therapy for PCME. None of the successfully treated cases have experienced a recurrence of PCME. Nevertheless, differences in intravitreal DEX implant therapy for PCME have been seen within these cases, as demonstrated in Figure 2.

### 3.3. Intergroup Differences in Efficacy between DEX Implant Alone and DEX Implant Plus Bevacizumab in PCME

Five patients underwent additional therapy with bevacizumab, while all other patients (*n* = 14) received DEX implants as monotherapy.

**Figure 3:**
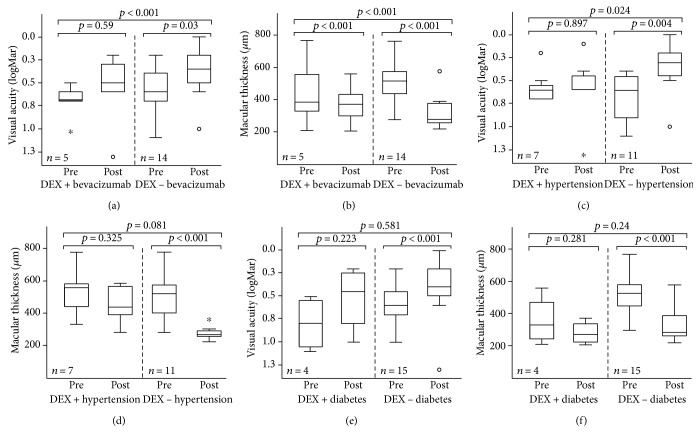
Visual acuity gain and macular thickness decrease comparison between DEX implantation therapy and combined DEX implantation and intravitreal bevacizumab therapy in PCME. (a) Visual acuity (logMAR) gain after DEX implant only compared to DEX implantation and bevacizumab injection is shown in this graphic. The box plot shows median visual acuity in logMAR (vertical line in the column), 95% confidence interval (column), and the standard deviation (extension lines) before and after treatment with DEX implantation in PCME patients groups. Statistical analysis with ANOVA followed by Tukey post hoc test for VA gain between patients treated with DEX implant only and DEX implantation and bevacizumab injection demonstrated a statistically significant higher increase of visual acuity in patients treated with DEX implant only (*p* < 0.001). (b) Central foveal thickness (*μ*m) reduction after DEX implant only compared to DEX implantation and bevacizumab injection is shown in this graphic. The box plot shows median central foveal thickness in *μ*m (vertical line in the column), 95% confidence interval (column), and the standard deviation (extension lines) before and after treatment with DEX implantation. Statistical analysis with ANOVA followed by Tukey post hoc test for central foveal thickness reduction between patients treated with DEX implant only and DEX implantation and bevacizumab injection demonstrated a statistically significant higher reduction of the central foveal thickness in patients treated with DEX implant only (*p* < 0.001). Visual acuity gain and macular thickness decrease after DEX implantation in PCME patients with and without arterial hypertension (c) Visual acuity (logMAR) before and after DEX implantation in PCME patients with and without hypertension is shown in this graphic. The box plot shows median visual acuity in logMAR (vertical line in the column), 95% confidence interval (column), and the standard deviation (extension lines) before and after treatment with DEX implantation in both PCME patients groups. Statistical analysis with ANOVA followed by Tukey post hoc test for VA gain between patients with and without arterial hypertension demonstrated a statistically significant higher increase of visual acuity in patients without arterial hypertension (*p* = 0.024). (d) Central foveal thickness before and after DEX implantation in PCME patients with and without hypertension is shown in this graphic. The box plot shows mean central foveal thickness in *μ*m (vertical line in the column), 95% confidence interval (column), and the standard deviation (extension lines) before and after treatment with DEX implantation. Statistical analysis with ANOVA followed by Tukey post hoc test for VA gain between patients with and without arterial hypertension demonstrated a higher, but not statistically significant decrease of the central fovea thickness in patients without arterial hypertension (*p* = 0.081). Visual acuity gain as well as macular thickness decrease after DEX implantation in PCME patients with and without diabetes (e) Visual acuity (logMAR) before and after DEX implantation in PCME patients with and without diabetes is shown in this graphic. The box plot shows median visual acuity in logMAR (vertical line in the column), 95% confidence interval (column), and the standard deviation (extension lines) before and after treatment with DEX implantation in both PCME patient groups. Statistical analysis with ANOVA followed by Tukey post hoc test for VA gain between patients with and without diabetes did not show a statistically significant difference of visual acuity increase in patients with and without diabetes (*p* = 0.581). (f) Central foveal thickness before and after DEX implantation in PCME patients with and without diabetes is shown in this graphic. The box plot shows mean central foveal thickness in *μ*m (vertical line in the column), 95% confidence interval (column), and the standard deviation (extension lines) before and after treatment with DEX implantation. Statistical analysis with ANOVA followed by Tukey post hoc test for VA gain between patients with and without diabetes did not show a statistically significant difference of visual acuity increase in patients with and without diabetes (*p* = 0.24). ° and ∗ symbolize single values with a 1.5- to 3-fold and above 3-fold interquartile range distance to the median.

Patients receiving only the DEX implant improved from a baseline VA of 0.60 (±0.23, max/min: 0.2 logMAR/1.0 logMAR) to 0.38 (±0.25, max/min: 0 logMAR/1.0 logMAR) logMAR after treatment (Figure 3(a)). This corresponds to Snellen equivalent baseline and postsurgery values of 20/80 and 20/50, respectively. A mean visual logMAR gain of 0.22 (±0.23) was reported for this group. Patients on combination therapy improved from a slightly worse baseline VA of 0.7 (±0.17, max/min: 0.5 logMAR/1.0 logMAR) to 0.58 (±0.37, max/min: 0.2 logMAR/1.3 logMAR) logMAR (Figure 3(a)). This corresponds to Snellen equivalent baseline and postsurgery VA values of 20/100 and 20/80, respectively. Visual gain following bevacizumab therapy was reported at a mean of 0.12 (±0.45) logMAR. The DEX implant-only treatment in PCME showed a significantly higher VA gain than the DEX implant/bevacizumab treatment (*p* < 0.001).

The mean baseline CFT in the DEX implant-only group improved from 501.57 (±128.82, max/min: 764 *μ*m/326 μm) to 307.64 *μ*m (±91.41 *μ*m, max/min: 574 *μ*m/210 *μ*m), while the combination group improved from 447.80 (±194.24, max/min: 767 *μ*m/310 *μ*m) to 372.20 *μ*m (±120.09 *μ*m, max/min: 560 *μ*m/276 *μ*m) (Figure 3(b)). The mean reduction in CFT in patients treated with DEX implant-only (*n* = 14) was 193.92 *μ*m (±14.65 *μ*m), while the mean CFT reduction in the DEX implant plus bevacizumab group (*n* = 5) was 75.60 *μ*m (±134.79 *μ*m). The DEX implant-only treatment in PCME showed a significantly higher CFT reduction than the DEX implant/bevacizumab treatment (*p* < 0.001). Therefore, a statistically significant mean CFT reduction was seen for DEX implant-only PCME patients.

### 3.4. Intergroup Comparison of Efficacy Results in PCME Patients with and without Hypertension

Patients without hypertension (*n* = 11) improved from a baseline VA of 0.67 (±0.25, highest VA: 0.4 logMAR, lowest VA: 1.1 logMAR) to 0.35 (±0.25, highest VA: 0 logMAR, lowest VA: 1 logMAR) logMAR after intravitreal DEX (Figure 3(c)). This corresponds to Snellen equivalent baseline and postsurgery values of 20/80 and 20/50, respectively. Patients suffering from hypertension (*n* = 8) improved from a baseline VA of 0.57 (±0.17, max/min: 0.2 logMAR/0.7 logMAR) to 0.59 (±0.34, max/min: 0.1 logMAR/1.3 logMAR) logMAR after intravitreal DEX (Figure 3(c)). Both values correspond to the Snellen equivalent value of 20/80. The logMAR mean VA gain was only 0.01429 (±0.26) in the subgroup of patients with hypertension, while the subgroup of patients without hypertension had a mean VA gain of 0.27273 (±0.273333) logMAR. Mean VA gains in the nonhypertensive subgroup were significantly better (*p* = 0.026).

Mean baseline CFT in patients without hypertension improved from 489.64 (±141.43, max/min: 767 *μ*m/310 *μ*m) to 271.63 *μ*m (±42.07, max/min: 385 *μ*m/210 *μ*m) after intravitreal DEX, while the hypertensive group improved from 521.57 (±136.03, max/min: 767 *μ*m/356 *μ*m) to 452.14 *μ*m (±106.44, max/min: 560 *μ*m/276 *μ*m) (Figure 3(d)). The mean reduction of CFT in the hypertensive group (*n* = 8) was 69 *μ*m (±158.58; min/max: −190/213 *μ*m), while the documented mean CFT reduction in patients without hypertension (*n* = 11) was 218 *μ*m (±153). The difference between groups was not statistically significant (*p* = 0.091).

### 3.5. Intergroup Comparison for Efficacy Results in PCME Patients with and without Diabetes

Patients without diabetes (*n* = 15) improved from a baseline VA of 0.58 (±0.19, max/min: 0.2 logMAR/1.0 logMAR) to 0.41 (±0.30, max/min: 0 logMAR/ 1.3 logMAR) logMAR after intravitreal DEX (Figure 3(e)). This corresponds to Snellen equivalent baseline and postsurgery values of 20/80 and 20/50, respectively. Patients suffering from diabetes (*n* = 4) improved from a baseline VA of 0.8 (±0.25, max/min: 0.5 logMAR/1.1 logMAR) to 0.53 (±0.31, max/min: highest VA: 0.2 logMAR/1.0 logMAR) logMAR after intravitreal DEX (Figure 3(e)). This corresponds to Snellen equivalent baseline of 20/125 and postsurgery values of 20/63. The subgroup of patients with diabetes had a mean logMAR VA gain of 0.275 (±0.311), while the subgroup of patients without diabetes had a mean VA gain of 0.173333 (±0.302141) logMAR. The difference in subgroup values was not statistically significant (*p* = 0.631).

Mean baseline CFT in patients without diabetes improved from 522.73 (±135.40, max/min: 767 *μ*m/356 *μ*m) to 337.40 *μ*m (±108.58, max/min: 574 *μ*m/261 *μ*m) after intravitreal DEX, while the diabetic group improved from 355 (±130.19) to 276.75 *μ*m (±62.24) (Figure 3(f)). Mean reduction CFT in the diabetics group was 78 *μ*m (SD 103.33) compared to 185 *μ*m (SD 157.69) in patients without diabetes. The difference between groups was not statistically significant (*p* = 0.19).

## 4. Discussion

The findings from this retrospective study further support the hypothesis that the use of a DEX implant in PCME results in improvements of VA and reduction of CFT. Patients additionally receiving bevacizumab did not show superior efficacy outcomes compared to DEX monotherapy. The VA improvement was shown to be significantly greater in patients without hypertension; however, no difference in efficacy was found for CMT reduction. Efficacy results for diabetics did not differ significantly from those obtained for nondiabetics.

Studies in the current literature addressing the intravitreal use of DEX implant in patients suffering from PCME are limited. In case patients still suffer from PCME (a normally self-limited disease after topical and/or oral treatment) DEX implant is a therapeutic option. So far, a randomized, prospective, single-masked, controlled trial reported on the efficacy and safety of DEX implants in 315 patients with persistent ME (≥90 days) [16]. A subset of 41 patients suffering from either uveitis or PCME was analysed separately. From this subset, a total of 27 patients had PCME. Of these, 9, 10, or 8 patients were randomized to observation, 350 *μ*g DEX intravitreal implant or DEX implant, respectively [16]. The analysis of the total subgroup (uveitis and PCME) found that on day 90, 53.8% of patients treated with DEX implant and 14.3% of patients on observation had gained ≥10 letters in BCVA (*p* = 0.29). Even though the primary outcome parameters from this study are not directly comparable to our data, it should be recognized that a VA gain of 10 letters would correspond to 0.2 logMAR units [16].

In further studies, the recalcitrant baseline BCVA of nine DEX implant patients was 0.62 ± 0.15 logMAR. At the last visit (6-month follow-up), the mean BCVA was 0.37 ± 0.26 logMAR (*p* = 0.002). The mean change from baseline foveal thickness was 143.89 *μ*m (decrease value of 26%) at month six, respectively. The authors concluded that both mean BCVA and improved foveal thickness after treatment with DEX implant remained statistically significant throughout the interval of six months [17]. Increase in VA gains was more pronounced in our study, while overall mean reduction in CFT was less. Almost similar findings have been seen in a group of 12 PCME patients treated with DEX implant [18].

A further retrospective review reported on the efficacy and safety of DEX implants over a mean period of 8.7 months in 14 PCME patients, including five diabetic patients [19]. The mean baseline VA was 0.72 logMAR, and mean preinjection CRT was 598 *μ*m [19]. The VA improved to 0.6 logMAR at month 12, respectively [19]. A second injection was necessary in eight patients, after a mean period of five months [19]. Even though patients in this study had worse VA and CRT thickness values at baseline, the overall improvement in VA rose comparably, but the decrease in CRT was less pronounced in our dataset.

A prospective case series in six diabetes patients, all of whom had developed PCME, treated consecutively with DEX implants reported a mean increase of 14 letters in BCVA from baseline to day 180 [20]. From baseline, the mean reduction of central subfoveal thickness achieved was 72 *μ*m by day 180 (*p* = 0.004) [20]. The VA improvement in this study appears to be slightly more pronounced than in our study [20].

A single case study documented DEX treatment in a patient with refractory PCME. This patient had been diagnosed with PCME 15 months earlier and pretreated with subtenon triamcinolone and intravitreal ranibizumab [21]. After the last injection with ranibizumab, the CFT was 640 *μ*m and BCVA was 78 letters. There was a complete remission after the first injection of DEX implant until day 187, when a second injection of DEX implant was administered [21]. Again, the edema resolved and VA was restored [21]. This gain of 6 letters corresponds to a logMAR gain of 0.1 and is less than what we saw in our population [21].

A prospective, nonrandomized, interventional case series compared the efficacy and safety of DEX implants versus intravitreally applied triamcinolone acetonide (IVTA) in 43 diabetics with PCME over six months [22]. While the DEX implant only had to be applied once, patients receiving IVTA had to be treated repeatedly (up to five times) After six months, 33% of patients in the DEX implant and 36% in the IVTA group achieved an improvement of a minimum of 10 letters [22]. The authors concluded that the DEX implant is a promising new therapeutic option in diabetics suffering from PCME, but in contrast to our study, diabetic retinopathy patients were not excluded [22].

While our data suggests that applying anti-VEGF treatment in addition to a DEX implant may not result in any additional efficacy, the following small studies consider the use of anti-VEGF compounds as monotherapies is efficacious.

The efficacy of intravitreal ranibizumab in PCME was reported as part of a retrospective data evaluation in seven patients. The authors found that there was a statistically significant difference in BCVA and CRT values before and after ranibizumab injection [23].

Demirel and colleagues reported ranibizumab to be an effective treatment in two patients suffering from PCME [24]. At the 21-month visit, BCVA had improved from 5/100 and 5/10 to 6/10 and 8/10, respectively, compared to baseline, while CMT was reduced significantly [24].

Diaz-Llopis and colleagues reported on the bevacizumab treatment of a single patient with refractory PCME (postintravitreal triamcinolone treatment). After one injection, the VA improved from 20/200 to 20/60. At the same time, the OCT showed a significant reduction in CRT [25].

The efficacy of 1.25 mg bevacizumab injections in PCME was reported in four patients [26], of which three showed improved VA [26]. In these patients, CRT also decreased to normal [26]. A further retrospective case series reported on bevacizumab treatment over a median duration of 14 weeks in 16 patients with PCME [27]. While VA improved by two EDTRS lines in one patient, it remained unchanged in 12 patients and decreased by two lines in two patients [27]. Repeated injections did not result in a better outcome [27].

Information on the combined treatment of a DEX implant with anti-VEGF compounds is still scarce. Fenicia and colleagues reported on a single case treated with ranibizumab followed by two injections of DEX implant for PCME. This strategy induced a progressive reduction of PCME until completely normal function was improved [28]. While this study used both therapies consecutively, a subpopulation from our study received both actives at the same time [28]. Therefore, the datasets are not comparable.

Our pilot study has some limitations that require discussion. The layout of a retrospective study always includes a selection bias. The number of patients screened is normally higher than the sample finally chosen for inclusion. The small sample size and geographic aspect represent a further limitation. The limitations listed above can be assumed for all retrospective data referenced above. Apart from one randomized controlled clinical trial, all datasets discussed were also neither randomized nor controlled. The treatment decision especially for an intravitreal administration of a DEX implant alone or with combined bevacizumab had not been clearly defined and there might be a negative bias for the decision to give additive bevacizumab. Also, the different group size of DEX implant alone or with combined bevacizumab weakens the significance of the data. Even more, DEX implant with combined bevacizumab was more frequently used in PCME patients with diabetes.

Overall current literature findings on intravitreal administration of a DEX implant in combination with VEGF inhibitors are scarce. Reports on anti-VEGF inhibitors alone are mostly of a retrospective nature and report only very small patient numbers. The data available on DEX implants seems to be slightly more robust.

The data from our study are in line with other reports on the efficacy of DEX implants, even though the different ways of reporting VA make direct interstudy comparability difficult. Treatment success factors are mandatory for any individual therapy planning, aim, and characteristic of modern medicine these days. Our data and other studies have taught us that a DEX implant has a benefit in PCME, but not in all patients. Therefore, we have investigated aspects, which might be a predictive factor for successful treatment with intravitreal DEX. Interestingly, we have seen a statistically significant lower VA gain of a DEX implant in patients with arterial hypertension, but not a statistically significant lower CFT. A higher number in both groups might have displayed also a statistical significance for CFT between patients with and without arterial hypertension.

The use of DEX implants in PCME should be further investigated in larger patient populations to confirm our findings. More predictive factors should also be investigated to improve the options for an individual PCME therapy with a DEX implant.

## Figures and Tables

**Table 1:** Demographic and anamnestic data, follow-up times and safety findings.

Number	Eye	Gender	Age at cataract surgery	Femto- assisted cataract surgery	Implanted IOL	Further previous eye surgeries	Previous treatment	Beginning of symptoms after cataract surgery (days)
1	Right	Female	78	No	Polytech H10 ASP +23.0 dpt	None	Topical diclofenac/ oral acetazolamide	48
**2**	**Right**	**Female**	**80**	**No**	**n/a**	**Intravitreal avastin**	**Topical diclofenac**	**31**
3	Left	Female	62	No	Polytech AS61+28.5 dpt	None	Topical diclofenac/ oral acetazolamide	86
4	Left	Female	82	No	Polytech AS61+11.0 dpt	IOL repositioning	Topical diclofenac/ oral acetazolamide	16
**5**	**Right**	**Female**	**53**	**Yes**	**Polytech H10 ASP+20.5 dpt**	**None**	**Topical diclofenac/** **oral acetazolamide**	**63**
6	Left	Male	63	Yes	AMO Tecnis +21.0 dpt	None	Topical diclofenac/ local dorzolamide	46
**7**	**Right**	**Male**	**76**	**No**	**Asphina 409 MP 21.5 dpt**	**None**	**Topical diclofenac/** **local dorzolamide**	**17**
8	Left	Female	77	No	Polytech H10 ASP +25.0 dpt	None	Topical diclofenac/ oral acetazolamide	8
**9**	**Left**	**Female**	**68**	**No**	**STAAR KS-Xs+22.5 dpt**	**None**	**Topical diclofenac/** **oral acetazolamide**	**61**
10	Right	Female	65	No	Euromaxx N313+18.5 dpt	None	Topical diclofenac/ oral acetazolamide	93
**11**	**Right**	**Female**	**73**	**Yes**	**Euromaxx N313+22.5 dpt**	**None**	**Topical diclofenac/** **oral acetazolamide**	**7**
12	Left	Male	62	Yes	Asphina 409 MP +20.5 dpt	Vitrectomy	Topical diclofenac/ oral acetazolamide	36
**13**	**Right**	**Female**	**76**	**No**	**Asphina 409 MP+20.5 dpt**	**None**	**Topical diclofenac/** **local dorzolamide**	**46**
**14**	**Left**	**Male**	**67**	**Yes**	**ZXRoo+23.0 dpt**	**None**	**Topical diclofenac/** **oral acetazolamide**	**35**
15	Left	Female	75	No	Euromaxx AL1313Y +23.5 dpt	None	Topical diclofenac/ oral acetazolamide	55
**16**	**Left**	**Male**	**78**	**No**	**Polytech H11+23.0 dpt**	**Focal argon laser coagulaton**	**Topical diclofenac/** **oral acetazolamide**	**84**
**17**	**Right**	**Male**	**62**	**No**	**Asphina 409 MP 20.0 dpt**	**None**	**Topical diclofenac/** **oral acetazolamide**	**14**
18	Right	Male	61	No	n/a	None	Topical diclofenac	16
19	Right	Female	64	No	n/a	IOL sulcus implantation + vitrectomy	Topical prednisolone/ oral prednisolone	42

The table summarizes patient parameter as well as cataract surgery specific parameter from all 19 patients included in this study. All patients who experienced a CFT reduction more than 200 *μ*m have been marked bold in this table.

**Table 2:** 

Number	Dexamethasone injection time duration after symptoms (days)	Effective phaco-emulsification time (sec)	Pretreatment central foveal thickness (*μ*m)	Δ logMAR (pretreatment/posttreatment)	Δ central fovea thickness (pretreatment/posttreatment)	Adjuvant bevanizumab injection (y/n)	Diabetes mellitus (y/n)	Arterial hypertension (y/n)	Follow-up time after Dex implant treatment (days)	Ocular side effects	Systemic adverse side effects
1	106	2.17	384	0	−14	y	y	Y	706	None	None
**2**	**496**	**n/a**	**767**	**−0.2**	**−207**	**y**	**n**	**Y**	**196**	**None**	**None**
3	256	3.39	356	0.1	29	n	n	N	242	None	None
4	197	1.96	550	0.1	24	n	n	Y	147	None	None
**5**	**136**	**0.13**	**510**	**−0.2**	**−249**	**n**	**n**	**N**	**64**	**None**	**None**
6	136	0.04	310	−0.2	−5	y	y	N	34	None	None
**7**	**108**	**4.71**	**522**	**−0.2**	**−237**	**n**	**n**	**N**	**81**	**None**	**None**
8	104	3.22	323	−0.1	−38	n	y	N	265	None	None
**9**	**675**	**1.73**	**489**	**−0.1**	**−213**	**n**	**n**	**Y**	**71**	**None**	**None**
10	240	1.28	436	−0.4	−190	n	n	N	60	None	None
**11**	**129**	**0**	**764**	**−0.5**	**−485**	**n**	**n**	**N**	**76**	**None**	**None**
12	239	0	568	−0.1	−179	n	n	Y	60	None	None
**13**	**77**	**0.88**	**582**	**−0.2**	**−205**	**n**	**n**	**Y**	**67**	**None**	**None**
**14**	**52**	**0**	**553**	**−0.8**	**−256**	**y**	**y**	**n**	**13**	**None**	**None**
15	295	1.66	325	0.6	104	y	n	y	798	None	None
**16**	**95**	**1.88**	**646**	**−0.8**	**−430**	**n**	**n**	**n**	**256**	**None**	**None**
**17**	**62**	**0.66**	**573**	**−0.4**	**−320**	**n**	**n**	**n**	**61**	**None**	**None**
18	64	n/a	311	−0.2	−33	n	n	y	77	None	None
19	240	n/a	462	−0.1	−189	n	n	n	61	None	None

The table summarizes outcome measurements, treatment procedures, selected systemic disease, and safety findings of all 19 patients included in this study. All patients who experienced a CFT reduction more than 200 *μ*m have been marked bold in this table.

## References

[1] Irvine S. R. (1953). A newly defined vitreous syndrome following cataract surgery. *American Journal of Ophthalmology*.

[2] Gass J. D., Norton E. W. (1966). Fluorescein studies of patients with macular edema and papilledema following cataract extraction. *Transactions of the American Ophthalmological Society*.

[3] Lobo C. (2012). Pseudophakic cystoid macular edema. *Ophthalmologica*.

[4] Chu C. J., Johnston R. L., Buscombe C. (2016). Risk factors and incidence of macular edema after cataract surgery: a database study of 81984 eyes. *Ophthalmology*.

[5] Do J. R., Oh J. H., Chuck R. S., Park C. Y. (2015). Transient corneal edema is a predictive factor for pseudophakic cystoid macular edema after uncomplicated cataract surgery. *Korean Journal of Ophthalmology*.

[6] Loewenstein A., Zur D. (2010). Postsurgical cystoid macular edema. *Developments in Ophthalmology*.

[7] Arevalo J. F., Garcia-Amaris R. A., Roca J. A. (2007). Primary intravitreal bevacizumab for the management of pseudophakic cystoid macular edema: pilot study of the Pan-American Collaborative Retina Study Group. *Journal of Cataract and Refractive Surgery*.

[8] Henderson B. A., Kim J. Y., Ament C. S., Ferrufino-Ponce Z. K., Grabowska A., Cremers S. L. (2007). Clinical pseudophakic cystoid macular edema. Risk factors for development and duration after treatment. *Journal of Cataract and Refractive Surgery*.

[9] Federman J. L., Annesley W. H., Sarin L. K., Remer P. (1980). Vitrectomy and cystoid macular edema. *Ophthalmology*.

[10] Bito L. Z. (1974). The effects of experimental uveitis on anterior uveal prostaglandin transport and aqueous humor composition. *Investigative Ophthalmology*.

[11] Augustin A., Loewenstein A., Kuppermann B. D. (2010). Macular edema. General pathophysiology. *Developments in Ophthalmology*.

[12] Jampel H. D., Brown A., Roberts A., Koya P., Quigley H. (1992). Effect of paracentesis upon the blood-aqueous barrier of cynomolgus monkeys. *Investigative Ophthalmology & Visual Science*.

[13] Kent D., Vinores S. A., Campochiaro P. A. (2000). Macular oedema: the role of soluble mediators. *The British Journal of Ophthalmology*.

[14] Grzybowski A., Sikorski B. L., Ascaso F. J., Huerva V. (2016). Pseudophakic cystoid macular edema: update 2016. *Clinical Interventions in Aging*.

[15] Bertelmann T., Witteborn M., Mennel S. (2012). Pseudophakic cystoid macular oedema. *Klinische Monatsblätter für Augenheilkunde*.

[16] Williams G. A., Haller J. A., Kuppermann B. D. (2009). Dexamethasone posterior-segment drug delivery system in the treatment of macular edema resulting from uveitis or Irvine-Gass syndrome. *American Journal of Ophthalmology*.

[17] Dutra Medeiros M., Navarro R., Garcia-Arumi J., Mateo C., Corcostegui B. (2013). Dexamethasone intravitreal implant for treatment of patients with recalcitrant macular edema resulting from Irvine-Gass syndrome. *Investigative Ophthalmology & Visual Science*.

[18] Klamann A., Bottcher K., Ackermann P., Geerling G., Schargus M., Guthoff R. (2016). Intravitreal dexamethasone implant for the treatment of postoperative macular edema. *Ophthalmologica*.

[19] Landre C., Zourdani A., Gastaud P., Baillif S. (2016). Treatment of postoperative cystoid macular edema (Irvine-Gass syndrome) with dexamethasone 0.7 mg intravitreal implant. *Journal Français d'Ophtalmologie*.

[20] Khurana R. N., Palmer J. D., Porco T. C., Wieland M. R. (2015). Dexamethasone intravitreal implant for pseudophakic cystoid macular edema in patients with diabetes. *Ophthalmic Surg Lasers Imaging Retina*.

[21] Brynskov T., Laugesen C. S., Halborg J., Kemp H., Sorensen T. L. (2013). Longstanding refractory pseudophakic cystoid macular edema resolved using intravitreal 0.7 mg dexamethasone implants. *Clinical Ophthalmology*.

[22] Dang Y., Mu Y., Li L. (2014). Comparison of dexamethasone intravitreal implant and intravitreal triamcinolone acetonide for the treatment of pseudophakic cystoid macular edema in diabetic patients. *Drug Design, Development and Therapy*.

[23] Mitropoulos P. G., Chatziralli I. P., Peponis V. G., Drakos E., Parikakis E. A. (2015). Intravitreal ranibizumab for the treatment of Irvine-Gass syndrome. *Ocular Immunology and Inflammation*.

[24] Demirel S., Batioglu F., Ozmert E. (2012). Intravitreal ranibizumab for the treatment of cystoid macular edema in Irvine-Gass syndrome. *Journal of Ocular Pharmacology and Therapeutics*.

[25] Diaz-Llopis M., Amselem L., Cervera E., Garcia-Delpech S., Torralba C., Montero J. (2007). Intravitreal injection of bevacizumab for pseudophakic cystoid macular edema resistant to steroids. *Archivos de la Sociedad Espanola de Oftalmologia*.

[26] Izdebski B., Michalewska Z., Dziegielewski K., Nawrocki J., Odrobina D. (2013). Treatment of cystoid macular edema with bevacizumab in course of Irvine-Gass syndrome. *Klinika Oczna*.

[27] Spitzer M. S., Ziemssen F., Yoeruek E., Petermeier K., Aisenbrey S., Szurman P. (2008). Efficacy of intravitreal bevacizumab in treating postoperative pseudophakic cystoid macular edema. *Journal of Cataract and Refractive Surgery*.

[28] Fenicia V., Balestrieri M., Perdicchi A., MauriziEnrici M., DelleFave M., Recupero S. M. (2014). Intravitreal injection of dexamethasone implant and ranibizumab in cystoid macular edema in the course of Irvine-Gass syndrome. *Case Reports in Ophthalmology*.

